# Hydrodynamic and Longitudinal Impedance Analysis of Cerebrospinal Fluid Dynamics at the Craniovertebral Junction in Type I Chiari Malformation

**DOI:** 10.1371/journal.pone.0075335

**Published:** 2013-10-10

**Authors:** Bryn A. Martin, Wojciech Kalata, Nicholas Shaffer, Paul Fischer, Mark Luciano, Francis Loth

**Affiliations:** 1 Conquer Chiari Research Center, University of Akron, Ohio, United States of America; 2 Department of Mechanical Engineering, University of Akron, Ohio, United States of America; 3 Spraying Systems Inc., Wheaton, Illinois, United States of America; 4 Mathematics and Computer Science Division, Argonne National Laboratory, Illinois, United States of America; 5 Department of Neurosurgery, Cleveland Clinic Foundation, Ohio, United States of America; University of Maryland, College Park, United States of America

## Abstract

Elevated or reduced velocity of cerebrospinal fluid (CSF) at the craniovertebral junction (CVJ) has been associated with type I Chiari malformation (CMI). Thus, quantification of hydrodynamic parameters that describe the CSF dynamics could help assess disease severity and surgical outcome. In this study, we describe the methodology to quantify CSF hydrodynamic parameters near the CVJ and upper cervical spine utilizing subject-specific computational fluid dynamics (CFD) simulations based on in vivo MRI measurements of flow and geometry. Hydrodynamic parameters were computed for a healthy subject and two CMI patients both pre- and post-decompression surgery to determine the differences between cases. For the first time, we present the methods to quantify longitudinal impedance (LI) to CSF motion, a subject-specific hydrodynamic parameter that may have value to help quantify the CSF flow blockage severity in CMI. In addition, the following hydrodynamic parameters were quantified for each case: maximum velocity in systole and diastole, Reynolds and Womersley number, and peak pressure drop during the CSF cardiac flow cycle. The following geometric parameters were quantified: cross-sectional area and hydraulic diameter of the spinal subarachnoid space (SAS). The mean values of the geometric parameters increased post-surgically for the CMI models, but remained smaller than the healthy volunteer. All hydrodynamic parameters, except pressure drop, decreased post-surgically for the CMI patients, but remained greater than in the healthy case. Peak pressure drop alterations were mixed. To our knowledge this study represents the first subject-specific CFD simulation of CMI decompression surgery and quantification of LI in the CSF space. Further study in a larger patient and control group is needed to determine if the presented geometric and/or hydrodynamic parameters are helpful for surgical planning.

## Introduction

**Figure 1:**
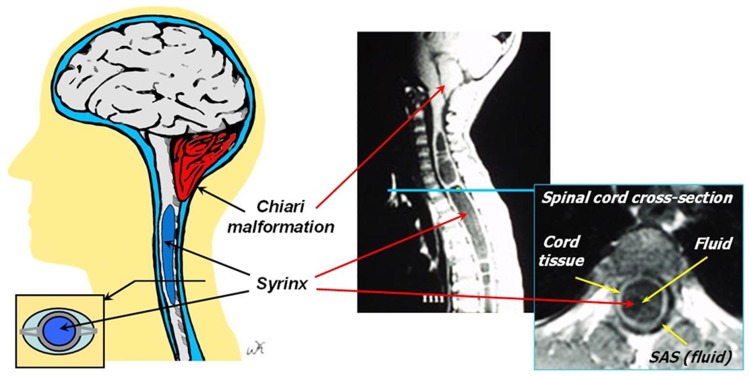
Simplistic representation and corresponding MR images (T1) of the CSF system around the brain and cervical spine for a Chiari patient with syringomyelia showing location of the syrinx (blue region in center of spinal cord) and tonsillar herniation (red at base of brain).

Alterations in cerebrospinal fluid (CSF) hydrodynamics at the craniovertebral junction (CVJ) have been suspected to play a role in the pathophysiology of Type I Chiari malformation (CMI) [1]–[7]. Historically, CMI has been anatomically defined by cerebellar tonsillar herniation below the foramen magnum (FM) of 5 mm or greater [8]. This measurement is typically made with a single sagittal plane T1-or T2-weighted MR image (Figure 1). Studies have sought additional morphometric measurements or combinations of measurements to help diagnose CMI [9], [10] and establish normal values [11]. These studies were relatively successful in differentiating CMI patients from healthy subjects. However, it has been shown that the standard herniation depth measurement does not necessarily correlate with neurological symptom severity [12] and patients with CMI-like symptoms have been found with less than 5 mm of herniation [13]. Also, a variety of morphometric measurements have been found insufficient to differentiate disease states related to CMI such as syringobulbia [14]. These difficulties may be due to the inability of static morphometric measurements to quantify dynamic aspects of the system such as CSF and neural tissue motion.

Researchers have sought to find an objective parameter that takes into account the dynamic nature of the system. One such parameter has been quantification of CSF velocity by phase contrast MRI (pcMRI). In a series of pcMRI studies, Haughton, et al. [1] and Quigley, et al. [2] found that peak systolic CSF velocity was higher in pre-surgery CMI patients than in healthy volunteers. Dolar, et al. [3] and Iskandar, et al. [4] showed that peak CSF velocity generally decreased after decompression surgery. In contrast, Sakas, et al. [6] and McGirt, et al. [15] found that peak CSF velocities were smaller in CMI patients than in healthy volunteers and increased post-surgery. Bunck, et al. [16], [17] utilized 4D PC MRI measurements to show elevated CSF peak velocities in CMI patients in comparison to healthy controls and abnormal CSF flow jets near the tonsillar stenosis. Thus, the dynamic quantification of CSF movement by pcMRI provides an objective dynamic measurement that may shed light on the pathophysiology of the CMI. However, more research is needed to understand why some results are conflicting.

An alternative technique to assess CMI dynamically is subject-specific computational fluid dynamics (CFD) simulation of the CSF motion. CFD can provide information about CSF dynamics that cannot be obtained directly by MRI such as: 1) highly detailed 3D information about the CSF flow field, 2) parametric alteration of boundary conditions to examine the specific impact of individual anatomical features or perturbations and 3) quantification of the pressure drop and other hydrodynamic parameters such as wall shear stress within the simulation region. A review of CFD simulations of CSF dynamics has been provided by Yiallourou, et al. [18] and Shaffer, et al. [7]. These studies focused on quantification of velocity and pressure drop as hydrodynamic parameters.

One hydrodynamic parameter that has not yet been used to quantify CSF dynamics is longitudinal impedance (LI). LI is the impedance to oscillatory flow per unit length of a conduit and its magnitude is a function of the conduit geometry and the mechanical properties of both the fluid and conduit. LI has most notably been used to investigate resistance to oscillatory flow in vein grafts. Using in vivo measurements, Schwartz, et al. [19] demonstrated that lower LI correlated strongly with primary graft patency. Meyerson, et al. [20] added that vein grafts with larger lumen diameters generally have more favorable impedance characteristics, but grafts with smaller lumen diameters may still demonstrate low LI, and thus better patency, when grafted. Moawad, et al. [21] showed that increasing the percent stenosis in ex-vivo vein grafts substantially increased LI despite only small changes in mean flow rate. This result was supported by Curi, et al. [22], who used an in vitro bypass graft flow model to show that LI magnitude remained relatively constant for a particular geometry, despite large changes in outflow resistance and flow rate. CSF outflow resistance has been studied by several of researchers [23], [24]. However, outflow resistance is mainly useful for understanding resistance to CSF drainage out of the cerebrospinal circulation and does not consider localized obstruction to CSF motion, such as the blockage created by cerebellar herniation in CMI. Because LI has demonstrated utility as a local measure of the impact of geometry on the flow field independent of systemic conditions, we suspect that it could be a useful parameter for quantifying the geometric complexity of changes to the cervical SAS anatomy, and hence severity of the blockage, in CMI.

Herein we describe a subject-specific hydrodynamic characterization of the upper cervical SAS in CMI patients both pre- and post- spinal decompression surgery in terms of LI and related CSF dynamic and geometric parameters. Our approach was to choose subjects with a variety of flow conditions for the study. Thus, we chose two 35-year-old symptomatic male CMI patients that were imaged both pre- and post- spinal decompression surgery and one 21-year-old healthy male volunteer (five cases were analyzed in total). Our objective was to compare the hydrodynamic parameters between each patient pre- and post-surgery and with the healthy control as a pilot study to understand the potential to assess hydrodynamic alterations between the groups.

## Materials and Methods

### Ethics Statement

MR data acquisition was performed at the Department of Radiology at the University of Illinois at Chicago. The study was approved by the institutional review board of the University of Illinois at Chicago. Prior to scanning, written informed consent was obtained from both the healthy volunteer and the CMI patients. All MR data was anonymized before being processed.

### MRI Measurements

Imaging for all five cases was performed on a 1.5-T GE Signa system (GE Medical Systems, Milwaukee, WI). Patients were evaluated for CMI using a standard T_1_-weighted anatomy scan in the sagittal plane and cerebellar herniation depth measurement similar to that described by Barkovich, et al. [25] Anatomy scans for the images used in model reconstruction were acquired using a vascular time-of-flight technique with T_2_-weighting and resulted in a set of transversely-oriented cross-sectional images of the lower cranial and cervical spinal SAS. Additional anatomical scanning parameters included slice thickness = 1.1–6.0 mm with no inter-slice spacing, FOV = 160–179 mm, and matrix size = 256×256. The healthy volunteer, a 21-year-old male (Healthy), was imaged from the lower cranial region to the bottom of the sacral spine. Two CMI patients, both 35-year-old males, were imaged both before (CP1-pre and CP2-pre) and after decompression surgery (CP1-post and CP2-post). The spinal anatomy of the CMI patients was imaged only from the FM to the lower cervical spine.

Phase-contrast MRI (pcMRI) images were acquired at the C2 level in all five cases using a 2D time-of-flight technique with retrospective peripheral pulse gating and VENC optimized for each subject ranging from 5 to 9 cm/s. Additional scanning parameters included TR = 17–22 ms, FA = 20–25°, FOV = 14–16 cm, matrix size = 256×256, and slice thickness = 5–6 mm [5]. Each pcMRI scan yielded a set of 32 images over the cardiac cycle.

### 3D Geometry and Meshing

A single operator performed the 3D reconstruction of the subarachnoid space (SAS) from the FM to the mid-cervical spine using MIMICS (Materialise, Ann Arbor, MI). The model reconstruction methodology consisted of thresholding the T_2_-weighted images for gray-scale range, masking the SAS, and manual editing to remove regions that did not communicate directly with the SAS (i.e. epidural space outside of the dura). The fine anatomical structures, such as the nerve roots, denticulate ligaments, and blood vessels, were not included in the geometric reconstruction as these structures were too small to be resolved. All SAS models were then converted to StrataSys Layer (SSL) slice-based format for geometric analysis and finite volume meshing.

**Figure 2:**
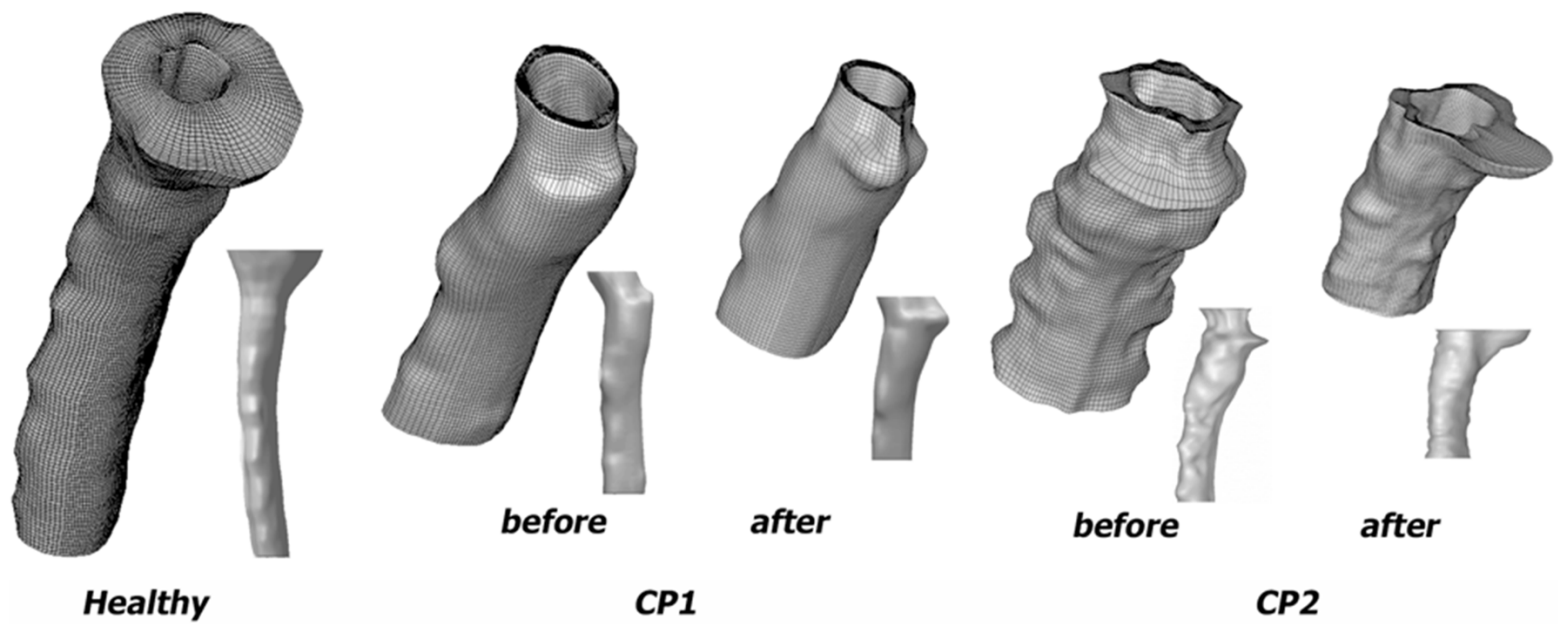
Meshed 3D reconstructions (sagittal view) of the cervical spinal SAS geometries used for the CFD simulations: healthy volunteer and two CMI patients before and after decompression surgery. Note: flow extensions used for the CFD simulations were not included in this figure.

The inner and outer surfaces of the SAS models were smoothed using a custom FORTRAN algorithm to remove non-physical features (i.e. roughness and sharp protrusions) of the anatomy resulting from pixilation artifacts. Details of the smoothing algorithm were published previously by Yedavalli, et al. [26]. The computational grid for each model was created using a custom MATLAB (The Mathworks, Natick, MA) algorithm. Details of the meshing algorithm were published previously by Lee, et al. [27]. To summarize, the meshing algorithm created an equal number of vertices on the inner and outer boundaries of each slice of the annular SAS model that were used to form hexahedral volume mesh cells. The program also created a file with mesh commands for STAR-CD (Adapco, London, England). All five meshed SAS geometries are shown in Figure 2.

### Flow Waveforms

Subject-specific CSF flow waveforms were used as boundary conditions for each CFD simulation in order to simulate subject-specific velocity fields. Each flow waveform was computed based on the pcMRI images obtained at the C2 level of each subject during the period of one cardiac cycle. Using MATLAB, a custom graphic-user interface (GUI) was developed to obtain flow waveforms by manually masking the SAS in each image set. Pixels were included or excluded from the mask based on visual verification of a coherent and pulsatile velocity profile. Subject-specific volume flow waveforms were then calculated from the summation of the velocity profiles multiplied by the area for each pixel in the mask.

### CFD simulation

Fluid flow simulations were performed using STAR-CD. All SAS geometries were treated as a rigid, having a blunt unsteady velocity inlet boundary condition (IBC) at the caudal end of the model, constant pressure boundary condition (CPBC) at the cranial end, and no-slip wall conditions at the inner (spinal cord) and outer boundaries (dura). Flow extensions were added to both the IBC and CPBC sides to reduce numerical instabilities in the flow field within the anatomic section of the flow domain. IBC/CPBC extension lengths were 5.8/9.7 cm, 4.3/2.5 cm, 6.2/3.4 cm, 4.0/5.3 cm, and 4.8/6.6 cm for the healthy, CP1-pre, CP1-post, CP2-pre, and CP2-post cases, respectively. As the mechanical properties of CSF and liquid water are similar, density and viscosity of the working fluid were specified as 1000 kg/m^3^ and 0.001 N·s/m^2^, respectively.

Each IBC was reconstructed from the respective volume flow waveform using Fourier methods. Cases were simulated for at least four cycles of the flow waveform to ensure that the results were cycle-independent. The MARS (Monotone Advection and Reconstruction Scheme) numerical method was used to solve the flow field with 0.005 s as the time step. Grid independence was tested on the healthy volunteer geometry by refining mesh node spacing until a repeatable solution was obtained. As all models were of a similar length scale, it was assumed that the same node spacing that produced a grid independent solution in the test model was sufficient for node spacing in the remaining models. Table 1 indicates the mesh and time parameters for each simulation. Post-simulation data processing and visualization was performed using STAR-CD and MATLAB.

**Table 1:** CFD simulation information for all cases.

Case	Number of Mesh Cells	Period Length (s)	Time Simulated (s)	Cycles Computed
Healthy	348800	0.834	3.84	4.6
CP1-pre	235200	1.071	5.43	5.1
CP1-post	206400	0.984	4.93	5.0
CP2-pre	153600	0.855	3.42	4.0
CP2-post	227600	0.780	3.12	4.0

### Geometric Parameters

Based on the 3D reconstruction and meshing, the following SAS geometric parameters were calculated and compared for all five cases: 1) cross-sectional area (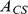) and 2) hydraulic diameter (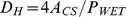). Axial distributions of 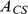 and 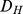 were obtained for each case by analyzing each axial cross-section defined in the respective SSL file.

### Hydrodynamic Parameters

The following hydrodynamic parameters were calculated and compared for all five cases: a) Reynolds number based on 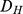 (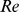), b) Womersley number based on 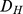 (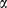), c) velocity at peak systole and diastole, d) unsteady pressure drop, and e) longitudinal impedance (Z_L_).

Reynolds number based on 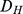 in each cross-section along the length of each model was computed by the relation 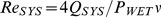, where 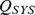 was the volume flow rate at peak systole and ν was the kinematic viscosity of CSF. Womersley number, 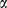, in each cross-section was computed by the relation 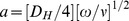, where 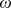 was the angular frequency of the volume flow waveform. Maximum velocity in the SAS at peak systolic (caudal direction),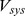, and diastolic flow (cranial direction), 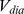, were identified in the velocity field from the CFD simulation.

The distribution of Reynolds number at peak systole, or the ratio of steady inertial forces to viscous forces, was utilized to assess the validity of the assumption of laminar flow (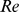
<2300) throughout the anatomical domain. Similarly, the distribution of the Womersley number, or the ratio of unsteady inertial forces to viscous forces, was used to assess the importance of inertia on the flow field, which was found to be large relative to viscous forces by Loth, et al. [28]. Further, the Womersley number indicates the changes in resistance along the length of the model.

The unsteady pressure drop across the craniospinal junction, 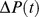, was computed as the difference in average pressure over the cross-sectional plane located at the FM, 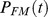, and average pressure at the cross-section at a plane located 2.5 cm caudal to the FM, 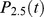, where 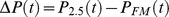. Studies have reported that tonsillar herniation >2.5 cm are rarely observed [8]. Hence, it was assumed that 2.5 cm in the longitudinal direction would encompass the entire region of the SAS where the geometry could be affected by tonsillar herniation.

LI was calculated by the ratio of Fourier coefficients of the pressure drop, 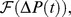 and flow waveforms, 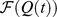, at each harmonic and then calculating an impedance modulus, 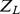, for each frequency, where 
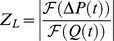
. The resulting curves for 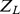 (in dyn-s/cm^5^) were then integrated from 1–8 Hz to obtain an LI value, 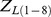, for each subject (in dyn/cm^5^). The frequency range of 1–8 Hz was selected because the harmonics of 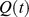 were small above 8 Hz in all cases. Thus, there was little contribution of frequencies greater than 8 Hz to fluid displacement. The zero-eth harmonic was not included as the focus of this study was on unsteady resistance. The above methodology is based on a similar calculation performed in vein graft patency studies by Meyerson, et al. and Skelly, et al. [29], [30]. A similar method has also been employed to understand the hemodynamic impact of proximal aortic grafts [31].

## Results

**Figure 3:**
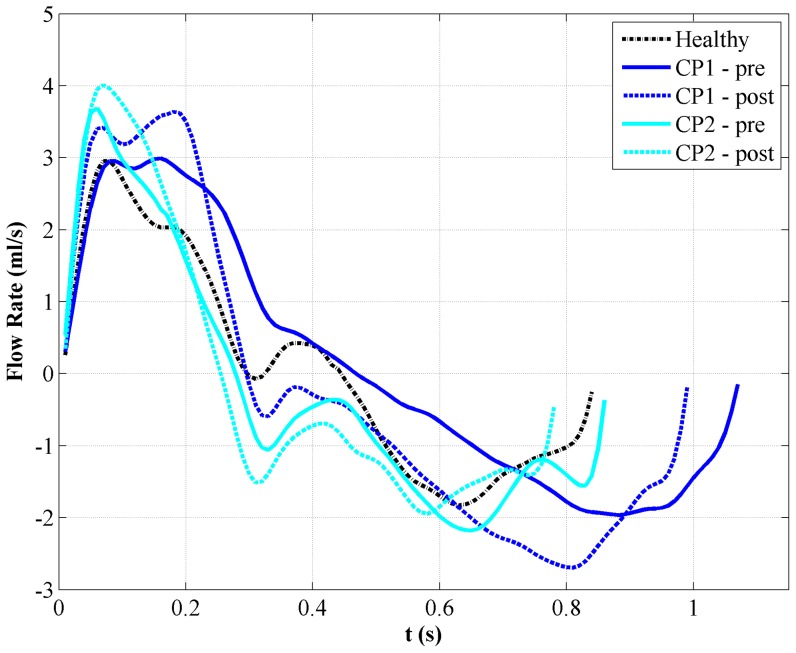
CSF flow waveforms at C2 for each case (Q represents flow). Note, positive flow is in the caudal direction and negative flow is in the cranial direction.

Results are presented for the healthy subject (Healthy) and two Chiari patients both pre and post surgery (CP1-pre/CP2-pre and CP1-post/CP2-post). From analysis of C2 flow waveforms measured by pcMRI, the magnitudes of peak systolic flow for the healthy volunteer, CP1-pre, CP1-post, CP2-pre and CP2-post cases were 2.95, 3.00, 3.63, 3.67, and 3.9 ml/s, respectively. The magnitudes for diastolic peak flow were 1.83, 1.95, 2.70, 2.18, and 1.92 ml/s, respectively (Figure 3). With the exception of CP1-pre, the waveform shape of all the measurements had similar features in terms of presence of a dicrotic notch during the deceleration phase and overall profile changes. It should also be noted that the CP1-Post, CP2-Pre, and CP2-Post cases had steeper acceleration from the initial zero-crossing in addition to higher peak systolic flow rates.

### Geometric parameters

**Figure 4:**
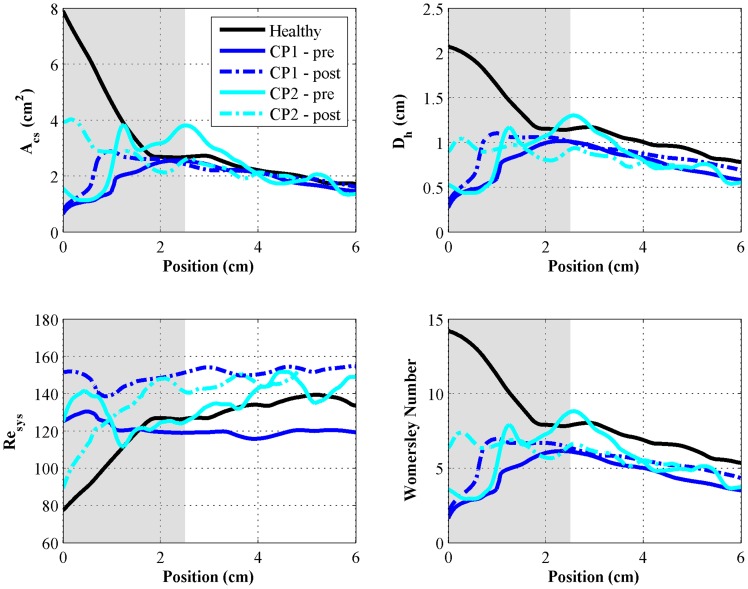
Distributions of hydrodynamic parameters computed for each case. Position is relative to the location of the foramen magnum moving caudally down the SAS. Highlighted segment of waveforms indicate values in the first 2.5

As expected, mean 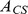 and 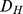 increased pre- to post-surgery for both CMI cases. Trends in the distributions of both 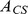 and 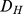 were similar for the CP1-pre, CP1-post, and CP2-pre cases; both parameters had a relatively sharp increase from a minimum value near the cranial end and then tapered gradually (Figure 4). In contrast, distributions of 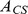 and 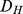 in the CP2-post and Healthy geometries both showed only a taper from a maximum value at the cranial end. Mean values of 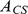 were 6.0, 1.2, 2.0, 2.4, and 2.9 cm^2^ for the healthy, CP1-pre, CP1-post, CP2-pre, and CP2-post cases, respectively. Mean values of 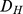 were 1.84, 0.52, 0.77, 0.82, and 0.92 cm for the healthy, CP1-pre, CP1-post, CP2-pre, and CP2-post cases, respectively.

### Hydrodynamic parameters

The aforementioned trends for 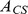 and 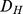 were nearly identical for the distribution of Womersley number for each case; values of 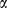 were comparatively low in regions of the CP1-pre, CP1-post and CP2-pre models with SAS narrowing caused by the CMI. Mean values of 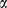 in the first 2.5 cm of each model were 12.7, 3.1, 4.9, 5.6, and 6.5 for the healthy, CP1-pre, CP1-post, CP2-pre, and CP2-post cases, respectively. Maximum Reynolds number was 111, 130, 152, 142, and 148 for the healthy, CP1-pre, CP1-post, CP2-pre, and CP2-post cases, respectively (Table 2 and Figure 4).

**Table 2:** Geometric and hydrodynamic parameters for each cervical SAS model.

Case	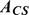 (cm^2^)	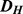 (cm)	Max 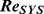	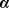
Healthy	6.0±1.2	1.84±0.18	111	12.7±1.3
CP1-pre	1.2±0.4	0.52±0.14	130	3.1±0.8
CP1-post	2.0±0.8	0.77±0.29	152	4.9±1.8
CP2-pre	2.4±1.0	0.82±0.30	142	5.6±2.0
CP2-post	2.9±0.6	0.92±0.07	148	6.5±0.5

Note: 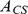, 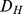, and 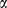 are mean values (Mean ± SD) calculated for the first 2.5 cm of the model length. Max 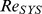 was also calculated for the first 2.5 cm of the model length.

**Figure 5:**
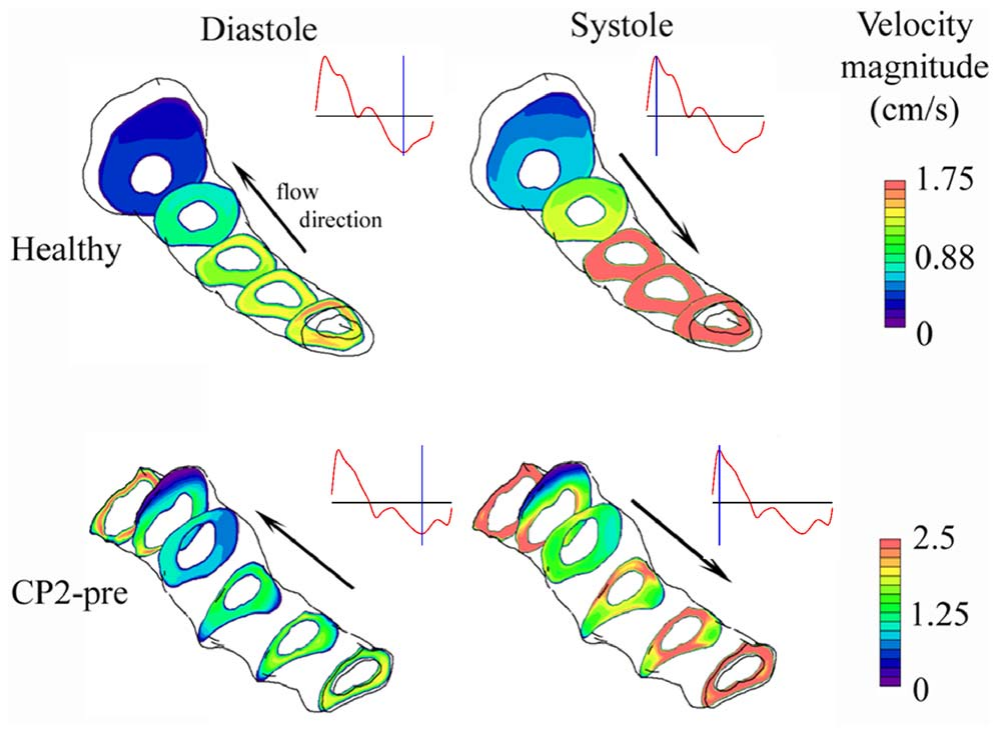
CSF velocity magnitudes simulated by CFD at various axial cross-sections shown at diastolic and systolic peak flow for the Healthy and CP2-pre case. Inset of flow waveform (red trace) indicates portion of CSF flow cycle that each plot is given (blue vertical line).

In the CMI cases, the maximum velocities, 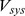, and 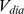, decreased pre- to post-surgery. The maximum velocities were located near the region of the SAS geometry affected by CMI (above the C2 level) for the CP1-pre, CP1-post, and CP2-pre cases. In contrast, peak velocities in the CP2-post and Healthy cases were located near the caudal end of the model (Figure 5), where geometric tapering was greatest (see trend of 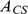 and 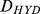 in Figure 4). For the healthy subject, velocity profiles were relatively uniform around the spinal cord over the entire model. For the patients, elevated CSF velocities were noted near the FM on the anterior side of the spinal cord in comparison to the posterior (see Figure 5 CP2-pre for an example).

**Figure 6:**
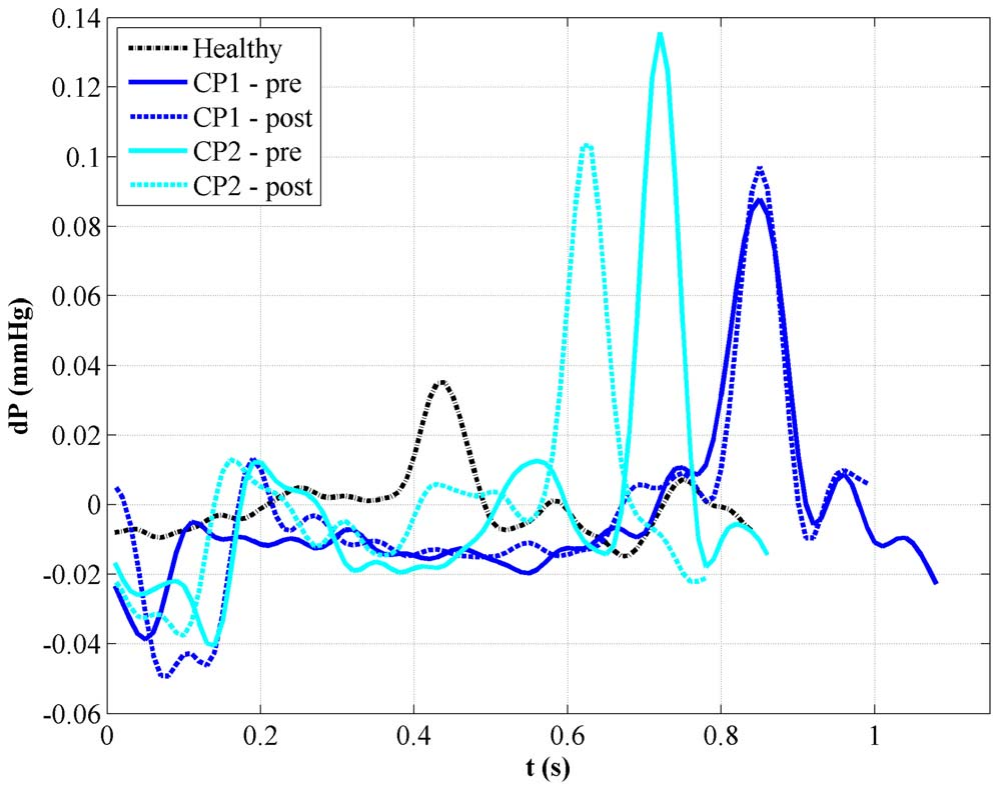
Pressure drop (dP) in the caudal direction computed over the first 2.5 cm of each CFD model.

The unsteady pressure drop, 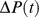, had a great degree of variation for the subjects examined (Figure 6). Peak pressure drop, 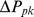, ranged from a maximum of 0.136 mmHg in CP2-pre to a minimum of 0.035 mmHg in the Healthy case (Table 3). 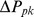 was at least 2× greater for the patients in comparison to the healthy subject (Table 3) and the timing of 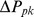 occurred earlier in the healthy subject than in the patients by 200 to 400 ms (Figure 6). 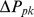 decreased pre- to post-surgery for the CP2 case, but increased slightly for the CP1 case.

**Table 3:** Maximum velocities and pressure drops.

Case	Max 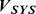 Peak (cm/s)	Max 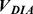 Peak (cm/s)	Region for Max 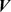	Max Δ P (mmHg)
Healthy	2.65	1.74	∼ inlet	0.035
CP1-pre	8.57	4.99	∼ top, tonsils	0.088
CP1-post	7.10	4.75	∼ top, tonsils	0.097
CP2-pre	6.79	3.99	∼ top, tonsils	0.136
CP2-post	3.94	1.71	∼1 cm to inlet	0.103

**Figure 7:**
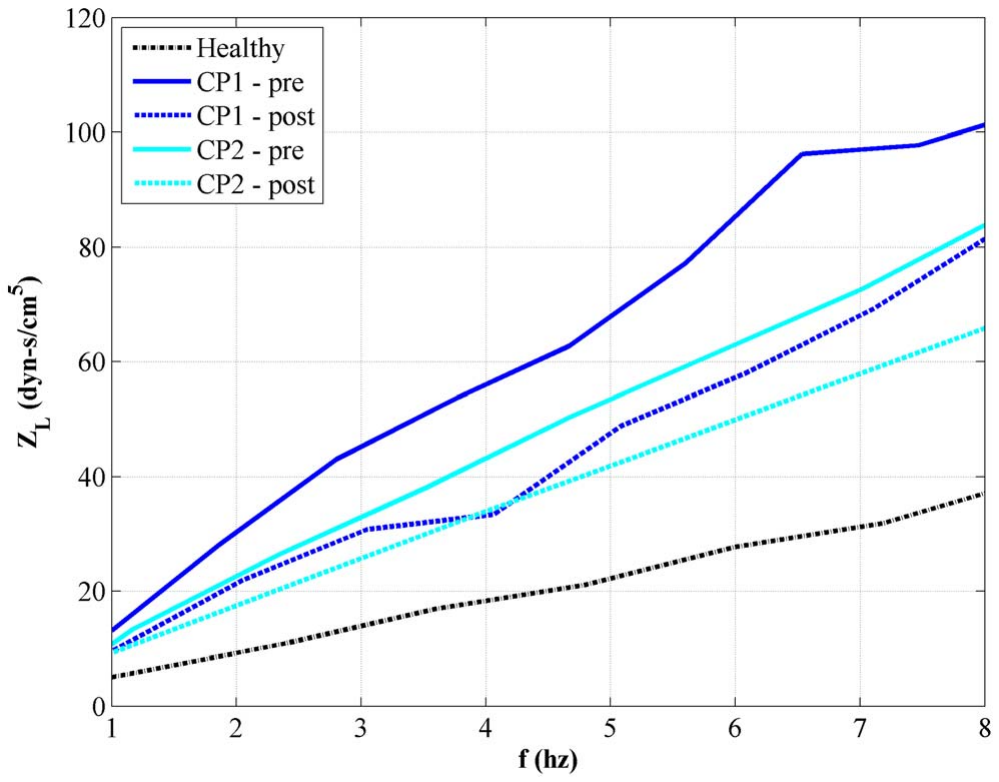
Frequency traces of longitudinal impedance values (Z_L_) for each case.

**Figure 8:**
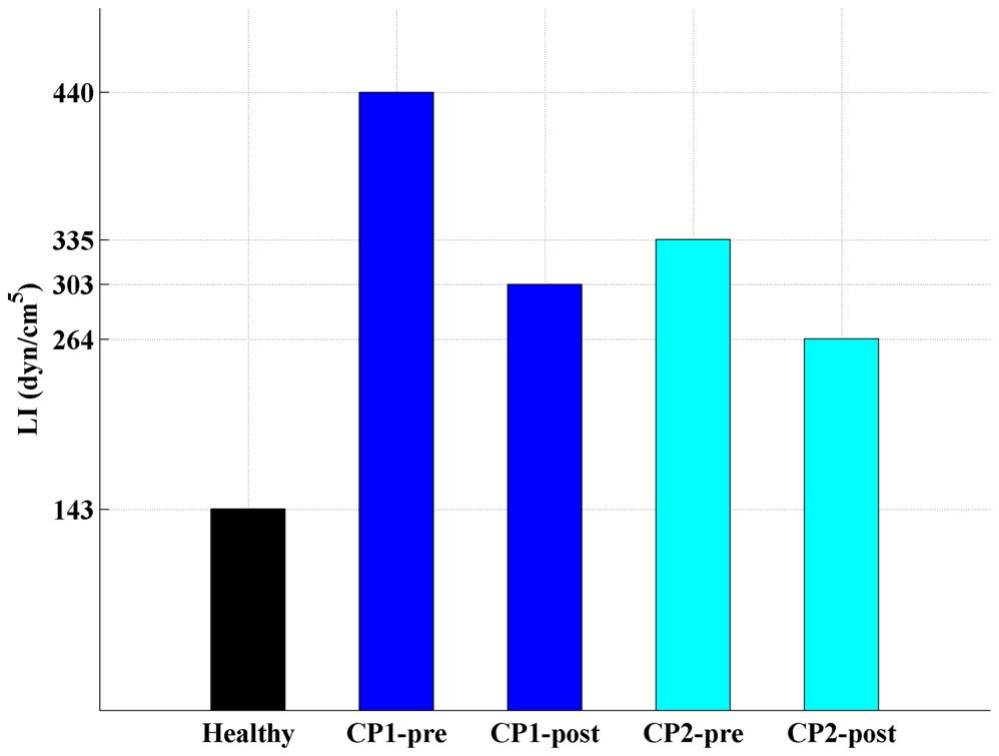
Integrated longitudinal impedance (LI) for each case.

LI was larger in patients than in the healthy subject than the patients and decreased following surgery (Figure 7 and Figure 8). For all five cases, the magnitude of the impedance modulus as a function of frequency, 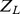, increased monotonically between 1 and 8 Hz (Figure 7). Integration of the impedance modulus from 1–8 Hz yielded an LI of 143, 440, 303, 335, and 264 dynes/cm^5^ for the healthy, CP1-pre, CP1-post, CP2-pre and CP2-post cases, respectively (Figure 8).

## Discussion

Altered CSF dynamics and SAS crowding at the CVJ have been thought to be an indicator of disease severity in CMI. However, CMI is a complex disorder that is likely due to many underlying conditions. As such, CSF dynamics and/or SAS crowding at the CVJ may not be important for all CMI patients, but rather a specific subset of CMI patients. This study provides methodology to quantify CSF dynamics in terms of the following hydrodynamic and geometric parameters: LI, maximum CSF velocity at peak systole and peak diastole, Reynolds and Womersley number, peak pressure drop, cross-sectional area, wetted perimeter and hydraulic diameter. These parameters were analyzed for two CMI patients pre- and post-surgery and one healthy subject by performing subject-specific CFD simulations for each case.

### Importance of the craniovertebral junction geometry

The geometric parameters quantified in this study, namely 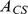 and 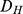, support the viewpoint that geometry of the CVJ is a critical aspect in CMI. In the limited number of data sets analyzed, the SAS geometry had the greatest variability at the CVJ within the SAS region from 0 to 2.5 cm caudal to the FM (Figure 2 and Figure 4). Caudal to the CVJ, the SAS geometry had little variation between the subjects analyzed. Decompression surgery impacted the SAS geometry to the greatest extent at the CVJ (Figure 4). However, geometric alterations to the CVJ varied for the two CMI patients analyzed. In both patients average CVJ 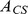 increased post-surgery. However, the axial variation of 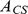 was different for each patient and even showed a decrease post-surgery for CP2 (2 cm caudal to the CVJ). This is surprising considering that this decompression surgery is conducted in order to expand area for CSF to move. The complexity of geometric changes between these geometric parameters is an indicator of the complex geometric alterations that can take place pre and post-surgery at the CVJ.

The axial variation in SAS geometry for the healthy and CP2-post cases resulted in similar values of 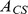 and 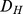 to those reported by Loth, et al. [28], where 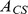 and 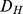 decreased in the cranio-caudal direction from the FM downward for the healthy subject (Table 2 and Figure 4). Loth, et al. [28] reported a maximum 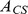 of 5 cm^2^, where the maximum 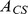 for the healthy and CP2-post cases in this study were 7.9 and 3.9 cm^2^, respectively. Surprisingly, to our knowledge, no studies in the literature have reported axial variation in SAS geometry in terms of 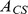 and 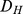 below the FM in both CMI patients and healthy controls. Figure 2 shows the great degree of 3D geometric differences that were present. Ideally, geometry of the SAS should be analyzed in a greater level of detail using 3D geometric analysis techniques and in a larger set of patients to understand their importance, if any, in CMI. However, as noted in the introduction, the MRI measurement technique used to quantify the geometric parameters was, by nature, static or time averaged over the cardiac cycle. Thus, the static geometric analysis presented could have limited utility if dynamic movement of neural tissue or CSF is an important factor.

### Hydrodynamic parameters and importance of dynamic analysis

Similar to the geometric parameters, the hydrodynamic parameters analyzed had a great degree of variability between the cases and the largest alterations were concentrated near the CVJ. These results support the consensus that CSF dynamics could be important in craniospinal disorders such as CMI.

Using both the diastolic and systolic peaks of the flow waveforms and hydraulic diameter, 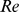 did not exceed 160 in any of the five cases (Figure 4), also consistent with previous findings [28], [32]. 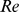 was significantly lower than the critical value for transition to turbulence and thus, we expect the flow to be laminar throughout the SAS, even in the patient cases analyzed where the SAS near the CVJ was constricted. Also, 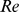 in CP1-pre, CP1-post, and CP2-pre cases did not vary greatly (maximum axial variation of ∼30%). In contrast, the healthy and CP2-post cases showed a sharp decrease in 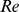 in the region immediately caudal to the cranium, due to larger hydraulic diameters (∼50% axial variation). Results for mean 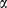 suggested that inertial effects were significantly dampened in the cervical SAS of each patient case compared to the healthy volunteer case and thus, resistance to the CSF pulsation was higher proximal to the CVJ. CP1 and CP2 showed post-surgical increases in proximal mean 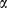 of 28% and 76%, respectively. However, post-surgical 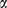 in both patient cases remained lower than that in the healthy volunteer.

In comparing the flow fields in healthy and patient cases (Figure 5), peak velocity and pressure drop (Table 3) in the CMI-affected geometries were higher, as expected. The relative changes in post-surgical peak systolic velocity were similar to those observed for 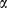 decreasing by 17% and 42% for the CP1 and CP2 cases, respectively. Surprisingly though, peak pressure drop increased 10% post-surgically for patient CP1 and decreased 24% for patient CP2, despite post-surgical increase in geometric parameters. However, it should be noted that geometric variability also increased post-surgically in patient CP1 in addition to the CP1-post flow waveform having a much steeper acceleration phase. Hence, differences in the flow fields were likely the result of differences in both geometry and the shape of the subject-specific flow waveform.

Similar to 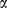, LI also showed large differences between the healthy control and both CMI patients. Likewise, LI in both CMI patients became closer to the level observed in the healthy control (Figure 7 and 8). Specifically, patient CP1 showed a 30% reduction and patient CP2 showed a 20% reduction in LI. However, post-surgical impedance in both CMI cases remained higher than the impedance observed in the healthy volunteer. This suggests that, though decompression surgery decreased LI, impedance was not restored to a “healthy” level. Tonsillar herniation was still present post-surgery that restricted CSF flow near the CVJ in comparison to the healthy subject (Figure 2).

LI was a parameter that 1) was different between the healthy control and both CMI patients and 2) became closer to the healthy control post-surgery for both CMI patients (Figure 7 and 8). The limited number of cases examined does not permit making generalizations, but for several reasons we believe LI shows promise as a tool to assess CMI severity and surgical outcome. Specifically, patient CP1 showed a 30% reduction and patient CP2 showed a 20% reduction in LI. However, post-surgical impedance in both CMI cases remained higher than the impedance observed in the healthy volunteer. This suggests that, though decompression surgery decreased LI, it was not restored to a “healthy” level. Tonsillar herniation was still present post-surgery that restricted CVJ CSF flow in comparison to the healthy subject (Figure 2).

### Limitations

Several limitations were present in the study and must be considered when interpreting the results. First, due to the limited number of subjects examined, it is not possible to deduce any general conclusions about the geometric or hydrodynamic parameters analyzed, but rather only potential for these parameters to assess disease states. Another limitation was that age and gender matching between the CMI patients and the healthy volunteer was not specifically controlled in recruiting subjects. Hence, some differences in spinal hydrodynamics may exist between the healthy subject and CMI patients. In the model reconstruction methodology, manual segmentation of the spinal SAS was employed. To reduce segmentation variability, the same operator segmented all models. Further, all images were obtained from the same MRI system and, thus, image quality was consistent. The spinal cord nerve roots, denticulate ligaments and blood vessels in the SAS were omitted in the segmentations. At present, these structures are too small to quantify with the current MRI resolution. However, the presence of these structures could potentially have an impact on the CFD flow field and hydrodynamic parameters such as pressure drop and LI. Validation of the CFD velocity field results was not possible because pcMRI was only obtained at the C2 level in our study. This could be accomplished in future work by comparing pcMRI at multiple planes or with 4D pcMRI conducted within the entire volume [18]. In the CFD analysis, a constant-pressure boundary condition was used at the FM. In reality, the pressure profile near the FM is unsteady. However, using an unsteady pressure boundary condition would not alter the resultant velocity field in a rigid-walled simulation. Lastly, the rigid-wall simulation did not account for any motion of the neural tissue that may occur over the cardiac cycle. Researchers have found that brain motion can be present in CMI patients [18], [33] and that a greater degree of tonsillar herniation could alter the flow field [34].

## Conclusion

Hydrodynamic characterization of CMI showed that decompression surgery can be quantified in terms of geometric and hydrodynamic parameters and that change in these parameters was concentrated near the CVJ. While these changes favored CSF motion at the CVJ, the parameters were not restored to the same level as in a healthy subject. CFD results were consistent with previously published clinical pcMRI studies that showed a decrease in peak CSF velocities in the CMI-affected cervical spinal SAS post-decompression surgery. LI was found to be a potential parameter to assess CSF blockage severity in CMI that includes system geometry and flow dynamics. Overall, the combination of MRI and CFD analysis provided detailed dynamic information about CMI that could lead to improved diagnostic tests for surgical planning and outcome assessment.
